# A self-assessment tool for predicting discomfort and tolerance in Chinese patients undergoing esophagogastroduodenoscopy

**DOI:** 10.1186/s12876-022-02364-0

**Published:** 2022-06-06

**Authors:** Jinqing Ou, Kuiqing Lu, Junzhen Li, Xi Deng, Junhui He, Guijin Luo, Hongdan Mo, Lingli Lu, Man Yang, Jinqiu Yuan, Pingguang Lei

**Affiliations:** 1 Department of Gastroenterology, Shenzhen Bao’an District Songgang People’s Hospital, No.2, Shajiang Road, Baoan District, Shenzhen, 518105 Guangdong China; 2 Clinical Research Center, Guangming District, The Seventh Affiliated Hospital, Sun Yat-Sen University, No.628, Zhenyuan Road, Shenzhen, 518107 Guangdong China; 3 Center for Digestive Disease, Guangming District, The Seventh Affiliated Hospital, Sun Yat-Sen University, No.628, Zhenyuan Road, Shenzhen, 518107 Guangdong China; 4 The First Clinical Medical School of Lanzhou University, No.1, Donggangxi Road, Chengguan District, Lanzhou, 730000 Gansu China

**Keywords:** Prediction, Cohort, Esophagogastroduodenoscopy, Comfort, Tolerance

## Abstract

**Background:**

For patients taking esophagogastroduodenoscopy (EGD), sedation should ideally be used individually based on patients’ comfort and tolerance level. However, currently there is no valid predictive tool. We undertook this study to develop and temporally validate a self-assessment tool for predicting discomfort and tolerance in Chinese patients undergoing EGD.

**Methods:**

We recruited 1522 patients undergoing routine diagnostic EGD without sedation. We collected candidate predictor variables before endoscopy and evaluated discomfort and tolerance with a 5-point visual analogue scale after the procedure. We developed logistic regression predictive models based on the first 2/3 of participants, and evaluated the calibration and discrimination of the models in the later 1/3 of patients.

**Results:**

30.2% and 23.0% participants reported severe discomfort or poor tolerance to EGD respectively. The predictive factors in the model for discomfort included sex, education, expected level of discomfort, and anxiety before endoscopy. The model for tolerance included income, expected level of discomfort, and anxiety before endoscopy. In the validation population, the established models showed a moderate discriminative ability with a c-index of 0.74 for discomfort and 0.78 for tolerance. Hosmer–Lemeshow test suggested the models had fine calibration ability (discomfort: P = 0.37, tolerance: P = 0.41).

**Conclusions:**

Equations for predicting discomfort and tolerance in Chinese patients undergoing EGD demonstrated moderate discrimination and variable calibration. Further studies are still required to validate these tools in other population.

***Trial registration*:**

Chinese Clinical Trial Registry (ChiCTR1800020236).

## Background

Esophagogastroduodenoscopy (EGD) is the gold standard test for the investigation of upper gastrointestinal symptoms, allowing direct view of mucosal surfaces, photography, biopsy, and therapeutic intervention [1]. EGD is universally accepted. In 2009, the total number of procedures performed in the U.S. alone reached approximately 6.9 million [2].

EGD is an invasive and unpleasant procedure which may trigger gag reflex, panic, fear, abdominal distention, and pain [3]. A survey of 509 patients suggested that 17.7% of the patients experienced severe discomfort during the procedure and 8% failed to complete the initial unsedated endoscopy [4]. Though the unpleasant experience can be effectively alleviated by sedation, sedation itself may lead to adverse events such as cardiorespiratory arrest, increased complexity and duration of endoscopy, and increased medical cost [5]. The sedation rate for EGD varied greatly across different countries. In North America and Australia, sedation is routinely applied for EGD [6, 7], while in Asian countries such as China and Japan, the use of sedation is often determined by patients’ preference, with an overall sedation rate of 50%-60% [7]. Given the benefits and harms of sedation, the ideal practice is not to apply sedation to all patients, but selectively to those patients at high risk of severe discomfort and poor tolerance.

Previous studies have observed that many factors, such as age [4, 8, 9], pharyngeal sensitivity [9], chronic use of psychotropic drugs or alcohol [10], and diameter of EGD [4] were associated with discomfort and tolerance in patients undergoing EGD. One of our recent studies also observed that the level of anxiety before endoscopy was independently associated with discomfort, tolerance, panic and fear during endoscopy in Chinese patients [11]. Findings of these studies suggested that it is possible to develop a predictive model based on readily acquirable data. However, current research is still limited. First, most previous studies aimed at investigating predictive factors, however a validated tool that could predict discomfort or tolerance with sufficient performance and applicability is not yet available. Secondly, effects of certain predictive factors were inconsistent among previous studies, for example, anxiety before EGD have shown mixed findings demonstrating either a harmful [4, 9, 10, 12] or null effect [8, 13–15]. Last, patient’s tolerance to EGD may be varied across countries, race, and cultures. While most previous studies were performed in Europe and North America, investigation of Chinese patients has been lacking. Thus, we undertake this study to develop and temporally validate a patient self-assessment tool for predicting discomfort and tolerance in Chinese patients undergoing EGD.

## Methods

### Design, study setting, and participants

This is a modeling development and validation study based on a prospective hospital-based cohort. We recruited 1522 inpatients or outpatients undergoing EGD from the Songgang Hospital (an upper second-class hospital in Shenzhen, China, with 900 beds and providing health services to 0.5 million local citizens) from May 2017 to April 2019. The sample size were estimated based on: 1) the estimated primary endpoint rate in the preliminary study (severe discomfort: 26%, poor tolerance: 19%); 2) the estimated number of predictive variables that will be included in the final models (5–10 for each model). We set the sample size to ensure the total number of case > 10 ✕ number of predictive factors for each model; 3) our workload. The inclusion criteria were as follows: 1) aged 18 years or above; 2) scheduled to undergo routine, diagnostic non-advanced EGD, for any reason; 3) received EGD without sedation; 4) undergoing EGD for the first time. We excluded patients with EGD contraindications, including pregnancy, esophageal stenosis, upper gastrointestinal tract anomalies, and a history of upper gastrointestinal surgery. We reported the study based on the transparent reporting of a multivariable prediction model for individual prognosis or diagnosis (TRIPOD) statement [16]. This study, including the consent procedure, was approved by the ethics committee of Songgang People’s Hospital (SGPHE201704G). The study was registered in the Chinese Clinical Trial Registry (ChiCTR1800020236, www.chictr.org.cn/showproj.aspx?proj=34010).

### Endoscopic procedure

Over 95% diagnostic EGD procedures are performed in the morning session of working hours in our hospital (8:00 am to 12:30 am), to avoid extensive fasting. Endoscopy nurses introduced the EGD procedure, benefits, and potential harms to participants before signing the informed consent form for endoscopy. All patients received lidocaine hydrochloride mucilage in a standardized fashion about 10 to 20 min prior to the procedure. Five certified endoscopists with at least three years of endoscopy experience performed all procedures. Patients were examined in the left lateral position with one of the endoscopes in our center (GIF-H260Z, GIF-Q260J, GIF-Q260, GIF-XQ260, Olympus, KeyMed, Southend-on-Sea, UK). The procedures were performed according to a standard operating procedure[17].

### Candidate predictor variables

We selected predictors that may influence the patients’ comfort and tolerance based on a review of previous studies [3, 4, 8–10, 14, 18–20] and discussion with endoscopists. Data were collected with a web-based questionnaire (WJX.CN) browsed through smart phones (https://www.wjx.cn/m/24278347.aspx). Participants were asked to finish the questionnaire independently. We provided assistance to those who required additional help, for example, the older people who were not familiar with smart phone. The covariates included sociodemographic characteristics (age, sex, weigh, height, education, and family income), lifestyle behaviors (smoking, alcohol drinking), recent use of psychotropic drugs (including antidepressant, antianxiety drug, antimanic drug, antipsychotic drug, tranquillizers, and others), diagnosis of diabetes, diagnosis of pharyngitis, recent pharynx- and larynx-related symptoms (sore throat, hoarseness, irritating cough, dysphagia, abnormal sensation of throat, dysphagia, and dry throat), expected level of discomfort regarding EGD (evaluated with a 5-point visual analogue scale (VAS)), self-evaluated personal tolerance level for uncomfortable feelings such as pain (5-point VAS), and current level of anxiety regarding the endoscopy (5-point VAS). Endoscopy nurses with at least 5-year working experience evaluated the pharyngeal sensitivity by the method described by Moulton et al. [21], and the view of oropharynx with Modified Mallampati Classification [22]. We also recorded the duration of the procedure and diameter of the endoscope after the procedure. We evaluated the content validity of the questionnaire for collecting predictors and the outcome data with a preliminary study of 200 patients and discussion with five gastroenterologists. Most of the questions were concise and straightforward and have been validated in previous study [15].

### Study outcome

In order to collect the outcome data, patients were asked to finish an online questionnaire (https://www.wjx.cn/m/24277153.aspx) independently about 10 to 20 min after endoscopy while waiting for the EGD report. Our endpoints included discomfort level (determined by asking ‘What is your level of discomfort during the procedure?’) and tolerance level (determined by asking ‘How hard do you feel to tolerate the discomfort during the procedure?’). Discomfort and tolerance level were evaluated with a 5-point VAS similar to previous studies [9, 10, 23], and were dichotomized based on a cut-off of 4 points (4 ≤ VAS score ≤ 5: severe discomfort / poor tolerance) in data-analysis.

### Data analyses

We carried out descriptive analysis and reported means and standard deviations (SD) for continuous variables and percentages for categorical variables. For the key predictors that are included in the final model, the missing value rate was generally less than 5%. We used multiple imputation to handle the missing data. Because the rates of ‘diabetes’ (20 cases, 1.57%) and ‘recent use of psychotropic drugs’ (25 cases, 1.26%) were too low, they were not included in the modeling analysis. For some categorical variables, small categories were collapsed when appropriate. For example, ‘past smoking’ (3.21%) was collapsed with ‘never smoking’, creating a ‘currently non-smoking’ category.

We developed predictive models with a multivariable logistic regression based on the first 2/3 of all included participants. For continuous variable, we used general additive model to investigate the potential non-linear association.[24] However, we did not find any continuous predictors that showed significant non-linearity. To select the predictive factors for final models, we used best subsets regression methods which compares the Akaike information criterion (AIC) of all possible models that can be created based upon the candidate predictors [25]. We evaluated the performance of the models based on the later 1/3 of included population. We evaluated the calibration by plotting the observed proportions of events against the predicted risks in 10 groups of equal size individual predicted risks, as well as by the Hosmer–Lemeshow test [26, 27]. We evaluated the discrimination of established models with concordance index (c-index).[27] We evaluated the explained variation in risk with R^2^ in the logistic regression[28]. Two-sided P < 0.05 was considered statistically significant for all analyses. Analyses were completed using R software version 3.4.1 (R Development Core Team, 2017).

## Results

### Baseline characteristics

This study finally included a total of 1522 eligible participants. We allocated the first 1015 participants to model development group and the later 507 patients to model validation group, according to the study protocol. The mean age in the model development group was 37.8 years and 38.6% were female. Most characteristics were similar in the two participant groups, except that the model validation group seems associated with a relatively higher rate of female (43.6% vs. 38.6%, P = 0.06) and normal pharyngeal sensitivity (93.3% vs. 88.6%, P = 0.02) (Table 1).

**Table 1:** Characteristics of participants for model development and validation

	Model development (N = 1015)	Model validation (N = 507)
Mean (SD) age, years	37.8 (10.3)	37.1 (10.2)
Female, n(%)	392 (38.6%)	221 (43.6%)
Mean (SD) BMI, kg/m^2^	22.1 (3.2)	21.8 (3.2)
Education		
Illiteracy, n(%)	50 (4.9%)	17 (3.4%)
Primary school, n(%)	97 (9.6%)	71 (14.0%)
Junior middle school, n(%)	445 (44.0%)	225 (44.4%)
Senior middle school, n(%)	252 (24.9%)	118 (23.3%)
University or higher, n(%)	168 (16.6%)	76 (15.0%)
Family annual income		
0–30 000 *Yuan*	416 (41.1%)	198 (39.1%)
30 000–80 000 *Yuan*	327 (32.3%)	185 (36.5%)
80 000–120 000 *Yuan*	149 (14.7%)	75 (14.8%)
120 000–300 000 *Yuan*	66 (6.5%)	33 (6.5%)
300 000 *Yuan and above*	36 (3.6%)	10 (2.0%)
Current smoker, n(%)	234 (23.2%)	101 (20.0%)
Current alcohol drinker, n(%)	169 (16.8%)	70 (13.9%)
Self-reported pharyngitis, n(%)	228 (22.5%)	94 (18.6%)
Current use of psychotropic medicine, n(%)	15 (1.5%)	4 (0.8%)
Mean (SD) self-evaluated tolerance^a^	3.1 (0.9)	3.1 (0.9)
Mean (SD) expected level of discomfort^a^	3.2 (0.9)	3.3 (0.9)
Mean (SD) level of anxiety before endoscopy^a^	2.7 (1.2)	2.7 (1.2)
Pharyngeal sensitivity		
Absent, n(%)	24 (2.8%)	8 (1.8%)
Attenuated, n(%)	75 (8.6%)	21 (4.8%)
Normal, n(%)	770 (88.6%)	406 (93.3%)
Mallampati classification		
Class I, n(%)	231 (26.6%)	121 (27.8%)
Class II, n(%)	301 (34.7%)	145 (33.3%)
Class III, n(%)	164 (18.9%)	78 (17.9%)
Class IV, n(%)	171 (19.7%)	92 (21.1%)
Indication of endoscopy		
* Healthy physical examination*	87 (8.6%)	42 (8.3%)
* Suspected gastrointestinal disease*	587 (57.8%)	288 (56.8%)
Gastrointestinal tumor warning symptoms	124 (12.2%)	77 (15.2%)
Various treatments under endoscopy	167 (16.5%)	83 (16.4%)
Lesions with regular follow-up	50 (4.9%)	17 (3.4%)
Endoscopy findings		
Chronic gastritis	980 (96.6%)	479 (94.5%)
Peptic ulcer	95 (9.34%)	53 (10.5%)
Gastric polyps	147 (14.5%)	76 (15.0%)
Esophagus-gastric varices	86 (8.5%)	38 (7.4%)
Gastric cancer	45 (4.4%)	20 (4.0%)
Portal hypertensive gastropathy	31 (3.1%)	16 (3.2%)
Esophagus-gastric submucosal tumor	99 (9.7%)	57 (11.2%)

*SD* standard deviation, *BMI* body mass index

^a^evaluated with a 5-points visual analogue scale

## Model development and validation

### Discomfort

A total of 308 participants (30.3%) in the model development group reported severe or extreme discomfort during EGD. Table 2 presents the associations of candidate predictors with discomfort. Univariate analyses suggested that age, sex, education, income, self-evaluated individual tolerance and anxiety before endoscopy were significantly associated with discomfort.

**Table 2:** Associations of candidate predictors with the primary outcomes

	Odds Ratio [95% Confidence Interval]			
	Discomfort		Tolerance	
	Univariate	Final multivariate model	Univariate	Final multivariate model
Age (continuous)	0.98 [0.96, 0.99]		0.97 [0.96, 0.99]	
Sex (female vs. male)	1.34 [1.02, 1.76]	1.22 [0.89, 1.68]	1.35 [1.00, 1.81]	
BMI (continuous)	1.01 [0.97, 1.05]		1.02 [0.97, 1.06]	
Education level				
Secondary school vs. primary school or illiteracy	2.01 [1.29, 3.15]	2.32 [1.42, 3.80]	1.69 [1.04, 2.74]	
Higher education vs. primary school or illiteracy	2.67 [1.58, 4.49]	2.65 [1.49, 4.70]	2.34 [1.33, 4.10]	
Family annual income				
30,000 – 300,000 Yuan vs. < 30 000 Yuan	1.15 [0.87, 1.52]		1.12 [0.82, 1.52]	1.19 [0.84, 1.70]
≥ 300,000 Yuan vs. < 30 000 Yuan	2.01 [1.01, 4.01]		2.09 [1.02, 4.29]	2.47 [1.07, 5.71]
Current smoker, (yes vs. no)	1.08 [0.79, 1.49]		0.95[0.67, 1.34]	
Current alcohol drinker, (yes vs. no)	0.91 [0.64, 1.30]		0.80 [0.55, 1.17]	
Pharyngitis, (yes vs. no)	1.01 [0.84, 1.23]		0.91 [0.74, 1.13]	
Snore, (yes vs. no)	0.98 [0.81, 1.19]		0.99 [0.80, 1.22]	
Pharynx and larynx related symptoms, (yes vs. no)	0.95 [0.72, 1.24]		1.09 [0.81, 1.47]	
Expected discomfort (continuous)	0.98 [0.84, 1.14]	1.38 [1.16, 1.65]	0.97 [0.82, 1.14]	1.14 [0.94, 1.38]
Self-evaluated personal tolerance (continuous)	1.59 [1.34, 1.87]		1.39 [1.16, 1.65]	
Anxiety before endoscopy (continuous)	1.85 [1.63, 2.10]	1.74 [1.51, 2.00]	2.32 [2.00, 2.69]	2.23 [1.90, 2.62]
Pharyngeal sensitivity (ordinal)	1.22 [0.77, 1.95]		1.03 [0.62, 1.70]	
Mallampati classification (ordinal)				
Class II versus Class I	0.87 [0.60, 1.26]		0.91 [0.61, 1.36]	
Class III versus Class I	0.74 [0.47, 1.15]		0.74 [0.45, 1.21]	
Class IV versus Class I	1.15 [0.76, 1.75]		0.87 [0.55, 1.39]	

*BMI* Body mass index

**Fig. 1:**
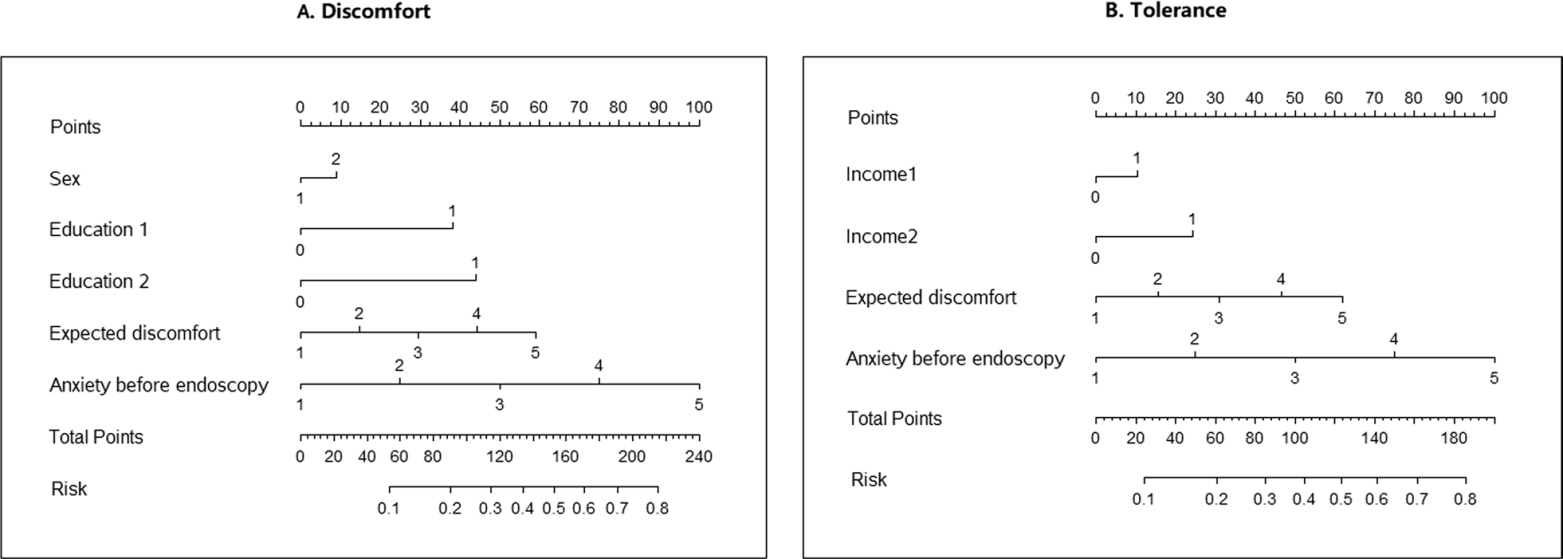
Nomogram for predicting discomfort and tolerance. Predictor values: Sex: *male* = 1*,female* = 2; Education1: *secondary school* = 1*, other* = 0; Education2: *higher education* = 1*, other* = 0; Income1: 30,000 – 300,000 *Yuan* = 1*, other* = 0; Income2: 300,000 *Yuan or higher* = 1*, other* = 0; Expected discomfort: *no discomfort* = 1*, mild discomfort* = 2*, moderate discomfort* = 3*, severe discomfort* = 4*, extreme discomfort* = 5; Anxiety before endoscopy: *no anxiety* = 1*, mild anxiety* = 2*, moderate anxiety* = 3*, severe anxiety* = 4*, extreme anxiety* = 5

We created the following model using best subsets regression: *Predicted probability* = 1/[1 + *exp*^(0.200×*sex* +0.842× *education*1+ 0.974× *education*2 + 0.336×*expected discomfort* + 0.552×*anxiety before endoscopy –* 4.451)^]*.* (Predictor values: Sex: *male* = 1*, female* = 2; Education1: *secondary school* = 1*, other* = 0; Education2: *higher education* = 1*, other* = 0; Expected discomfort: *no discomfort* = 1*, mild discomfort* = 2*, moderate discomfort* = 3*, severe discomfort* = 4*, extreme discomfort* = 5; Anxiety before endoscopy: *no anxiety* = 1*, mild anxiety* = 2*, moderate anxiety* = 3*, severe anxiety* = 4*, extreme anxiety* = 5). We developed a nomogram to predict the probability of discomfort (Fig. 1A).

**Fig. 2:**
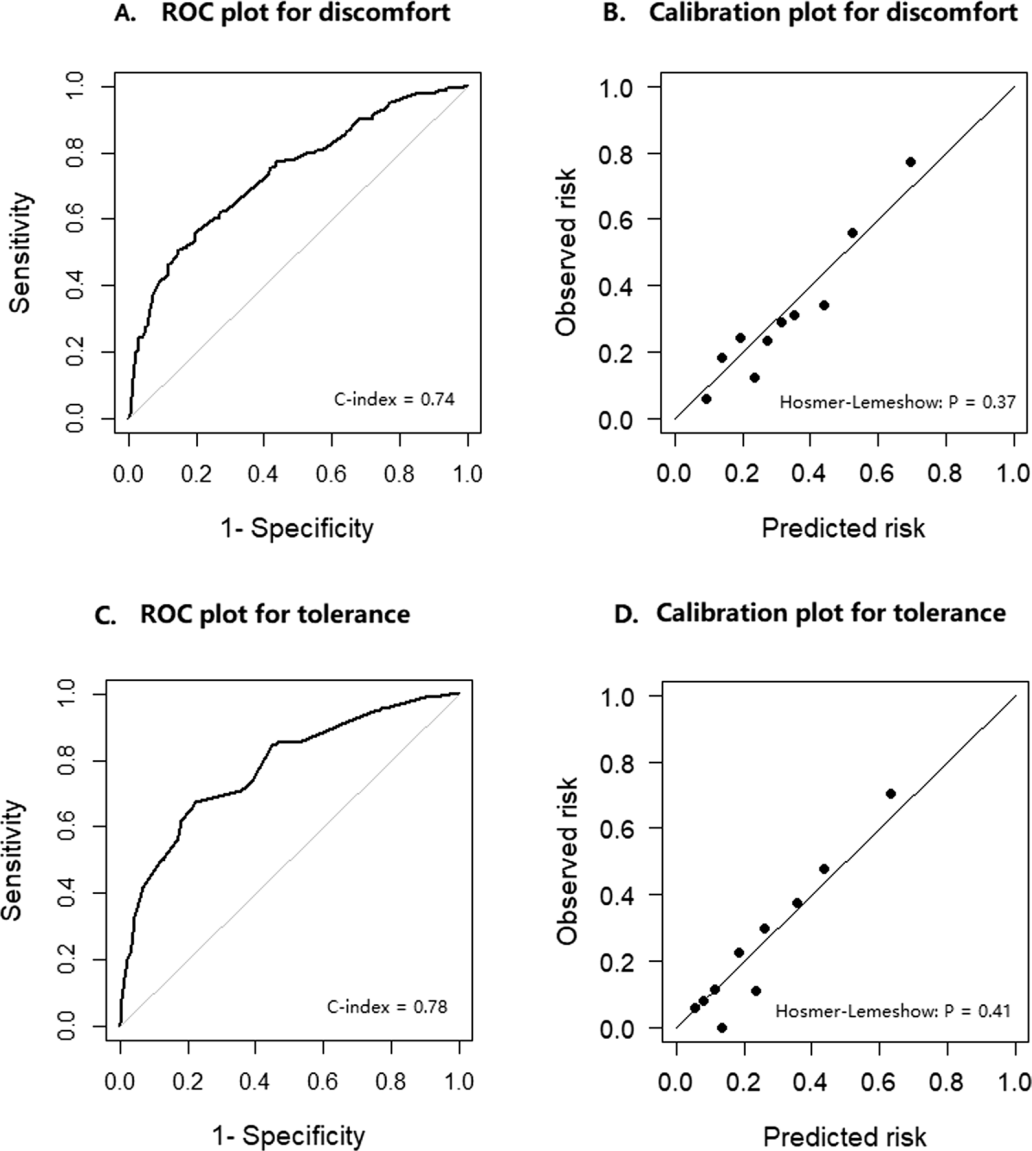
Receiver operating characteristic curve and calibration curves for discomfort and tolerance

There were 155 participants (30.5%) in the model validation population reported severe or extreme discomfort. The established model show a moderate discriminative ability with a C-index of 0.74 (95% confidence interval (CI) = 0.69 to 0.79) (see the ROC in Fig. 2A). The model showed fine calibration ability with no significant difference by Hosmer–Lemeshow test (P = 0.37, see the calibration plot in Fig. 2B).

### Tolerance

A total of 231 (22.7%) participants in the model development population and 119 (23.5%) in the model validation population reported poor tolerance. The final predictive model for tolerance was as follows: *Predicted probability* = 1/ [1 + *exp*^(0.178×*income*1+0.906×*income*2+0.131× *expected discomfort* + 0.802×*anxiety before endoscopy −* 4.136)^]*.* (Predictor values: Income1: 30,000* –* 300,000 *Yuan* = 1*, other* = 0; income2: 300,000 *Yuan or higher* = 1*, other* = 0; Expected discomfort: *no discomfort* = 1*, mild discomfort* = 2*, moderate discomfort* = 3*, severe discomfort* = 4*, extreme discomfort* = 5; Anxiety before endoscopy: *no anxiety* = 1*, mild anxiety* = 2*, moderate anxiety* = 3*, severe anxiety* = 4*, extreme anxiety* = 5*.*). The predicted probability can be calculated by the nomogram which was presented in Fig. 1B. In the model validation population, the predictive model for tolerance showed moderate discriminative ability with a C-index of 0.78 (95%CI = 0.72 to 0.83) (Fig. 2C). The model showed fine calibration ability with no significant difference by Hosmer–Lemeshow test (P = 0.41, Fig. 2D).

### Performance of the predictive models

To evaluate the performance of the predictive models, we estimated the sensitivity, specificity, and positive/negative predictive value at different cut-off points (Table 3). For example, if we classify the discomfort of participants based on a predicted discomfort risk ≥ 0.3, then 50% of the participant will be predicted as with severe or extreme discomfort, 72% patients actually with discomfort and 60% patients actually with no discomfort will be correctly predicted, 45% participants who are predicted to be with severe or extreme discomfort and 83% with no discomfort will be correctly predicted. As expected, the sensitivity and negative predictive value decrease with cut-off risk, while specificity and positive predictive value increase with cut-off risk.

**Table 3:** Performance of the predictive model for discomfort and tolerance at different cut-off points

Cut-off risk	% predicted case	Sensitivity	Specificity	LR +	LR-	PV +	PV-
Discomfort							
0.2	0.71	0.86	0.35	1.34	0.38	0.38	0.85
0.3	0.50	0.72	0.60	1.82	0.46	0.45	0.83
0.4	0.28	0.51	0.83	3.01	0.59	0.58	0.79
0.5	0.16	0.37	0.93	5.23	0.68	0.70	0.77
0.6	0.19	0.30	0.86	2.05	0.82	0.47	0.73
Tolerance							
0.2	0.55	0.85	0.55	1.88	0.28	0.38	0.92
0.3	0.27	0.57	0.83	3.30	0.52	0.52	0.86
0.4	0.22	0.50	0.88	4.05	0.57	0.57	0.84
0.5	0.10	0.29	0.96	7.06	0.74	0.70	0.81
0.6	0.08	0.23	0.97	7.02	0.80	0.69	0.79

*LR* + positive likelihood ratio, *LR* negative likelihood ratio, *PV* + positive predictive value, *PV* negative predictive value

## Discussion

In this hospital-based cohort of Chinese patients undergoing unsedated EGD, we developed two tools for predicting discomfort and tolerance. These models show moderate discrimination and fine calibration in a temporal validation cohort from same endoscopy center. Because the predictors of these models included readily available data including age, sex, education, family annual income, expected discomfort, self-evaluated personal tolerance, and anxiety before endoscopy, these models can be easily used by patients and clinicians.No extra tests or complex computations are required.

In a previous study of 148 patients, the investigators developed an instrument to predict satisfaction and poor tolerance based on nervousness before procedure and chronic use of alcohol or psychotropic medications [10]. In another study of 251 outpatients undergoing diagnostic EGD or colonoscopy, the investigators developed a model to predict patient satisfaction according to age, type of procedure, education, and anxiety scale [29]. A study of 336 Canadian patients developed a model for predicting patient comfort based on age and pharyngeal sensitivity [8]. However, the aforementioned models were not validated by appropriate methodology in those studies.

Anxiety before EGD is the most important predictor we found in our study, which was in agreement with a number of previous studies [4, 9–11, 29], Anxiety before EGD was strongly associated with all study outcomes, particularly for panic and fear during the procedure. The associations were reasonable as both anxiety before EGD and the outcomes were closely related to the emotional status of patients. The present study, for the first time, found that high expected discomfort and low self-evaluated personal tolerance were associated with poor patient-related outcomes. It’s expected that interventions for reducing anxiety before EGD may be beneficial for reducing discomfort and improving tolerance. Potentially effective interventions include oral midazolam [30], music [31], written educational material [32], behavioural intervention [33], and audio and visual distraction [34]. All these interventions have showed beneficial effects. However direct comparisons among these interventions are not available. As these interventions are not mutually exclusive, it is expected that a combined use of multiple interventions may have the maximum effect on anxiety.

We found higher education and income were associated with poorer patient outcomes. A possible explanation is that those patients are often at higher socioeconomic status, thus they have greater demand for painless subjective experience and feelings. In line with previous studies [3, 4], we also observed that females were associated with poorer EGD experiences than males. This could be explained that females were often more sensitive to pain and other unpleasant experiences than males [29]. The indications of ESD could be a factor that may influence the tolerance. In this study, the procedures were all basic EGD and were performed without sedation. Thus these procedures were similar in complexity and duration, which in turn did not increase the precision of prediction in our analysis.

To the best of our knowledge, the present study is the first study that developed and temporally validated clinical models for predicting patients’ experience for unsedated EGD. This is also the first modeling study for EGD experience in Chinese population. The applicability of these models is high because all predictors were based on readily available data such as sex and anxiety level. We created nomograms that could easily calculate the probability of unpleasant experience.

This study has limitations. First, the study population for model development and validation were recruited from the same hospital, so whether these models are applicable to other population is still unknown. Secondly, the pre-endoscopy anxiety, expected discomfort and self-evaluated tolerance can change overtime, so even for the same patient, the predicted discomfort or tolerance may not always be the same. Third, we did not include patients who had previously received EGD because they already had the first-hand experience, those who still chose unsadeneted EGD maybe more tolerable. It is still unknown whether our models are applicable for those with previous EGD experience.

## Conclusion

Our findings have important implications for clinical practice and future research. Although we have not validated our models in an external population, our results suggested that it is possible to personalize sedation for EGD based on the predicted discomfort and tolerance. The equations for predicting discomfort and tolerance in Chinese patients undergoing EGD demonstrated moderate discrimination and variable calibration. The evaluation can be performed easily and conveniently without demand for special equipment or tests. Further studies are still required to validate these tools in other Chinese population.

## Data Availability

The datasets used and/or analyzed during the current study are available from the corresponding author on reasonable request.

## Abbreviations

EGD: Esophagogastroduodenoscopy
TRIPOD: Transparent reporting of a multivariable prediction model for individual prognosis or diagnosis
VAS: Visual analogue scale
SD: Standard deviations
AIC: Akaike information criterion
CI: Confidence interval
